# Positional Manoeuvres for BPPV: Theoretical Approach to Remote Training for Non-specialists

**DOI:** 10.3389/fneur.2021.738785

**Published:** 2021-10-05

**Authors:** Vassilios Tahtis, Amanda Male, Diego Kaski

**Affiliations:** ^1^King's College Hospital NHS Foundation Trust, Therapies Rehabilitation and Allied Clinical Services, London, United Kingdom; ^2^Department of Brain Sciences, Imperial College London, London, United Kingdom; ^3^Therapy Services, National Hospital for Neurology and Neurosurgery, London, United Kingdom; ^4^Department of Clinical and Movement Neuroscience, Centre for Vestibular and Behavioural Neurosciences, Institute of Neurology, University College London, London, United Kingdom

**Keywords:** BPPV, Dix-Hallpike, epley, virtual, training

## Introduction

The COVID 19 pandemic resulted in the extraordinary transition of many aspects of healthcare to “telemedicine” based platforms (1). While this has been a long-recognised possibility in a variety of medical specialties including stroke care (2), it was the rapidity of this transition which has been particularly striking. A close, arguably less discussed parallel to the delivery of clinical care using remote platforms, is the training of healthcare staff utilising similar technologies. Digital transformation of information and knowledge is the most recent paradigm shift (3) in our society, increasingly embraced by healthcare and academic institutions. Here we consider the possibility of leveraging such technologies to address a common, treatable clinical presentation.

Benign paroxysmal positional vertigo (BPPV) is the commonest cause of dizziness among the general population. Its incidence is conservatively estimated at 64 per 100 000 population per year (4) and is more common among the elderly population (its prevalence approaching 9% in those >65 years of age) (5). Diagnosing BPPV is important because the symptoms can be disabling and yet the disorder is easily treated. In most instances, it is thought to be caused by calcium carbonate crystals (from the otolith organs) that settle within the endolymphatic fluid of one or more semicircular canals, where they do not belong. A history of recurrent brief episodes of spinning vertigo triggered by head movement suggests BPPV, but a definitive diagnosis lies on a positional manoeuvre which will elicit positional nystagmus in patients with the disorder. Box 1 highlights the main indications for a positional manoeuvre.

Box 1 **Indications for positional testing.**

- Any patient with brief episodic vertigo, especially positional vertigo, without spontaneous nystagmus.
- Patients with otherwise unexplained unsteadiness, particularly in the elderly (where there may be a dissociation between vestibular activation and vestibular perception).
- Individuals with attacks of non-positional vertigo are unlikely to have BPPV, but a positional manoeuvre can be worthwhile. If the positional manoeuvre elicits a typical BPPV-like nystagmus the patient should undergo repositioning, but if normal the patient should be referred onwards.

Given that BPPV may affect any one of the six semicircular canals in the head (three in each ear), one practical approach is to perform a Dix–Hallpike manoeuvre for right and left posterior semicircular canals as these are the most commonly involved (up to 95% of all BPPV cases (6). A manoeuvre such as the Dix-Hallpike should arguably be performed on every patient presenting with dizziness or imbalance because BPPV is common, carries an excellent treatment success rate, and dizzy symptoms are difficult for patients to describe (making history alone insufficient to make a confident diagnosis). Despite being an established procedure for the diagnosis and management of BPPV, positional manoeuvre are still substantially under-performed, mostly where it matters most: general practice and emergency settings, as this is where many patients with BPPV present (7). As such, there is an unmet need to improve training in positional manoeuvres across emergency, community, and primary care settings.

Assessment of the dizzy patient requires a comprehensive understanding of theory, examination and obtaining an appropriate patient history to exclude other causes of positional vertigo, nystagmus and more sinister pathologies. This degree of comprehensive assessment may be beyond the remit of non-specialists without more intensive training. Here, we focus specifically on positional manoeuvres for BPPV and explore aspects of a training program which may be amenable to the use of technologies or remote education. We argue that training therapists, not just physicians, is an important goal in ensuring BPPV is identified more promptly across emergency and primary care settings. In many services therapists already play a role in the assessment and treatment of BPPV, with development and access to telemedicine one possible avenue to increase the proportion of therapists who are competent and able to perform the associated manoeuvres. Communicating education through web-based technologies is commonplace–most notably, the use of video sharing platforms for interested individuals to self-direct their learning. While such platforms may contain excellent information, they potentially contain similar volumes of inaccurate and misleading content. We suggest the acceptance of web-based learning as convention provides the opportunity to develop comparative resources, scrutinised for rigour much the same way that a peer review process provides a degree of probity to the reader.

Constraints imposed during the COVID-19 pandemic saw clinical services pivot to digital technologies for a number of aspects of patient care. Here we consider if this accelerated implementation of telemedicine could meaningfully extend to training for BPPV, addressing a recognised shortfall of suitably trained healthcare staff. We describe some of the pitfalls to such telemedicine approaches, but also highlight practical factors that will increase the chances of a successful training programme and how existing technology may support this. We focus on the components which should comprise the training package for key positional manoeuvres for the diagnosis and treatment of common types of BPPV, namely Dix-Hallpike for the diagnosis of posterior semi-circular canal BPPV, and the Epley treatment manoeuvre.

## Components of Training

Components of a training program for practitioners learning assessment and treatment manoeuvres should involve both theory and practical components. Box 2 outlines what a typical training program may include. The specific components should follow recommendations from the Barany Society (8), the British Society of Audiology procedure guidelines (9), and expert opinion regarding educational requirements (10).

Box 2 **key components of a training program.**

BPPV Training:

- Theory
- Manoeuvres training
  - Operational details
    ∗ Room set-up
    ∗ Subject and examiner positions
  - Supervised practice
  - Identifying the correct ear
  - Pearls and Pitfalls
- Eye movement detection
  - Nystagmus types
  - Pattern of onset
  - Video bank

Clinical observations

The practical training poses perhaps the most overt challenge given the relative paucity of specialists with training expertise, and the need to cover a wide geographical distribution for sufficient uptake to make a tangible clinical difference at a population level. Remote learning in fact may offer a practical solution by pooling the scant resource of expert educators from almost any geographical location to provide training to a widely distributed audience of learners.

### Dix-Hallpike and Epley for Posterior Canal BPPV

**Figure 1:**
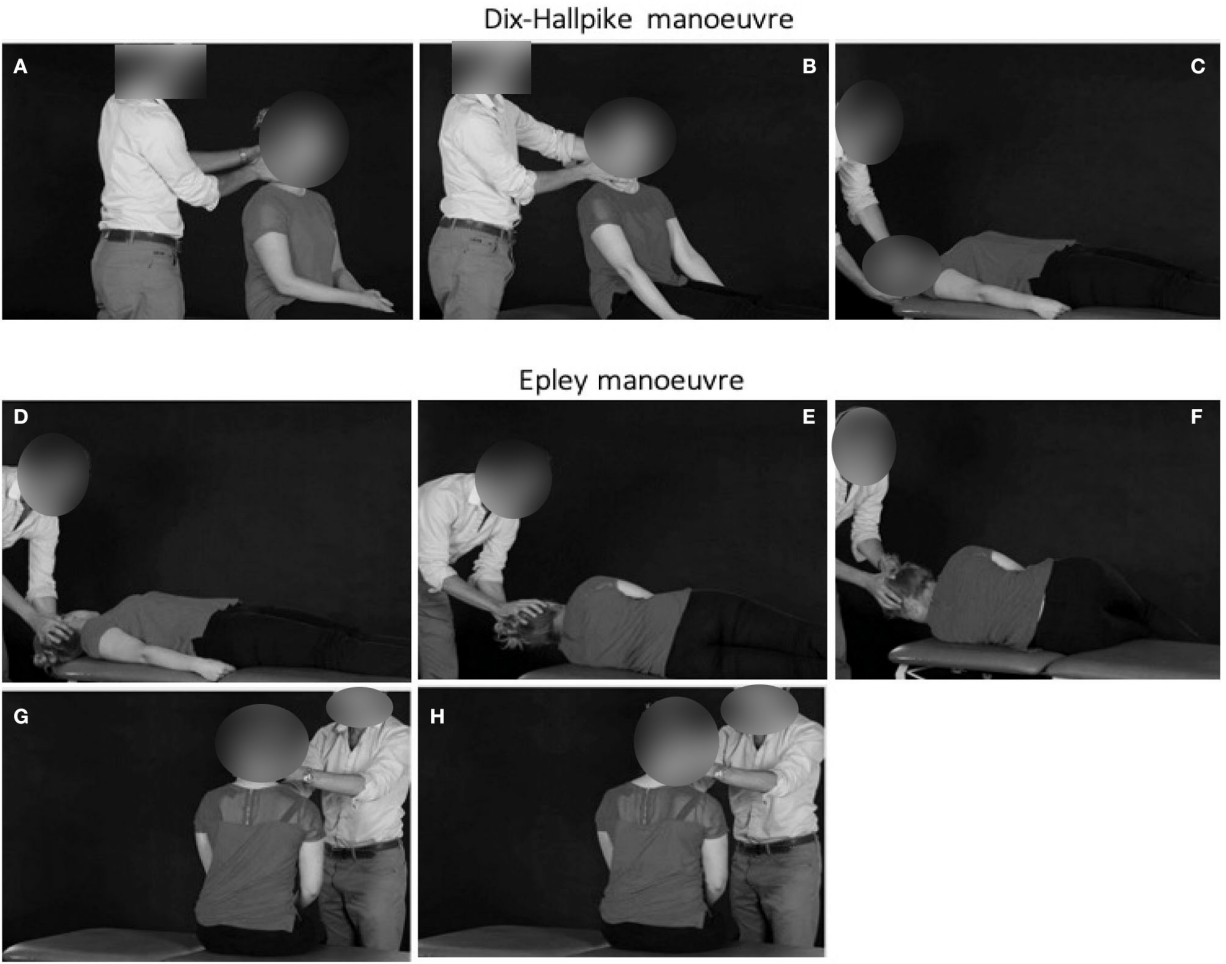
The first manoeuvre is the Dix–Hallpike manoeuvre exploring the right ear **(A–C)**. The patient begins seated in the couch, with the feet longitudinally. The head is then turned towards the ear being explored (right ear in this case). The patient is then lowered backwards such that the head is hyperextended by approximately 30°, and overhangs the end of the couch. In the presence of BPPV, there is upbeat and torsional nystagmus beating toward the ground with vertigo, usually lasting only 5–10 s. It may be necessary to open the eyes lightly using your thumb and index fingers to see the nystagmus more clearly. Epley manoeuvre for treating right ear posterior canal BPPV **(D–H)**. After 30 s, the patient's head is rotated toward the other side [leftwards here, **(D)**], and held in this position for a further 30 s. During this time, the patient is asked to rotate the body so that he/she is lying on their left shoulder **(E)**. It is important to hold onto the head while the patient is instructed to turn, so that it does not change position. The patient's head is rotated by a further 90° to the left, to face the ground **(F)**, and held for 30 s. The patient is now sat up, with the head still looking over the right shoulder **(G)**, for 30 s. Finally, the patient's head is brought toward the midline, and the neck flexed, chin down through 45° for a final 30 s **(H)**. The procedure is reversed for left posterior canal-BPPV. *Adapted from* (11).

The Dix-Hallpike manoeuvre is a simple bedside examination for the diagnosis of BPPV and can be performed with the patient placed longitudinally on the couch (Figures 1A–C). If BPPV is diagnosed on the Dix-Hallpike this lends itself to an Epley treatment manoeuvre (Figures 1D–H). If the history strongly suggests a symptomatic side, it may be best to test the non-symptomatic side first (and then do the manoeuvre on the other side) as it prepares the patient for the manoeuvre without inducing vertigo. The Epley manoeuvre is one standard treatment approach for posterior canal BPPV and involves 5 steps through which the patient's head (and body) is rotated on the couch. It requires space around the couch, but can be performed at a gentle pace, using gravity to guide movement of the offending inner ear crystals through the semicircular canal. These manoeuvres could be taught through remote teaching approaches, using video material and voice-over to guide the learner through the manoeuvre and identify common areas of difficulty or concern that learners may report. Such material could be complemented with virtual or online resources, such as a question-and-answer forum and guidance pertaining to pearls and common pitfalls for the novice non-specialist (Box 3).

Box 3 **Pearls and pitfalls for positional manoeuvres.**

Explanation of the manoeuvre in simple terms. Do not give too much information so as not to overwhelm the patient!

If utilising a traditional Dix-Hallpike, the manoeuvre uses gravity to mobilise the crystals within the posterior SCC, therefore it is performed with gentle slow movements.

If side-lying, the movement may need to be brisker to overcome gravitational forces, as the crystals are migrate upwards and around within the canal. You may need to get some help from the back, to hold the patient's shoulders. Ensure bed is raised appropriately to avoid injury to your back.

Patients experience heterogenous responses to Dix-Hallpike. It is common to have patients report intense vertigo, while others may deny any symptoms at all, with symptom subjectivity not always correlating to the severity of eye movements observed.

For both traditional and side-lying manoeuvres, test one side first, and the other if negative. If the first side tested produces symptoms and nystagmus, then go straight to the treatment manoeuvre.

It is unusual to have bilateral BPPV, so there is no need to test the other side after a treatment. If symptoms however persist over the coming days, it is good practice to re-test, starting with the side that was not initially treated.

Fatigability of the nystagmus can occur if the Dix-Hallpike is repeated frequently within a short timeframe. If a Dix-Hallpike is performed and the result is inconclusive, a period of 15-30 min should be allowed before retesting, with a maximum of two treatments in a single day.

Warn the patient about possible sudden unsteadiness and even risk of falls after the repositioning manoeuvre, and ensure the patient is supported immediately after the manoeuvre to avoid falls.

## Training Delivery Methods

Remote training can take many forms. In this context we are describing the use of pre-prepared content in the form of videos and lectures and the use of online seminars that allow a distributed network of interested parties to access content through web-based platforms. One critical distinction would be the need to validate such a training program, developed and updated by expert/specialist clinicians in a suitable position to adequately scrutinise the information and communicate it effectively. Using the framework outlined in Box 2, we propose a training program constituted of several components, the majority of which could be completed remotely.

Theory presents the most obvious and natural component of training which could be delivered remotely. Ideally, live lecture series could be delivered through video communication software by an expert to suitable trainees. Similar approaches have resulted in positive experiences and outcomes (12), with didactic interactions, recordings of the lecture and transcription presenting a notable advantage over conventional teaching approaches.

Manoeuvre training has conventionally been taught using face to face practical sessions and departures from this approach are ostensibly viewed as inferior. However, several approaches might be considered in combination. The use of high quality well produced video content, correctly narrated can provide repeatable demonstration of the correct techniques, to be revisited by the learner as required. Video sessions, with the instructor demonstrating manoeuvres in real-time using a healthy volunteer or mannequin would allow for immediate dialogue and clarification between students and instructor. Similarly, utilising supervised remote practice, where novice learners demonstrate the manoeuvres through real-time video sessions would allow for skilled experts to observe and provide feedback on technique. Another useful tool, easily adopted by remote approaches would be to provide video examples of patients during actual assessment and treatment. While such videos typically focus on interpreting eye movements (which we outline below), an equally important component is providing learning materials which developing an understanding of the heterogenous responses patients may have, particularly to testing. For instance, when patients develop intense symptomatic responses, the specialist will typically be well positioned and able to reassure patient sufficiently to complete the assessment, while having the experience to reassure them and remain unperturbed if the patient becomes nauseous or the assessment results in emesis. Similarly, many patients respond to testing by instinctively reaching to stop themselves being lowered to a plinth or may shut their eyes to alleviate dizziness. Videos which highlight such responses are likely to better prepare non-specialists and increase the likelihood of a successful manoeuvre.

Correct interpretation of eye movements and the pattern of nystagmus is crucial to successful assessment and treatment. Utilising a video bank of typical, expected responses to assessment (positive and negative) as well as common alternative observations provides a powerful resource for the developing practitioner. Such a resource could be expanded further, to include web-based assessments where students can practice correct interpretations and be tested on their performance and accuracy.

Having undertaken positional manoeuvre training there remains the important transition of taking the newly learned skills into the clinical setting. In the context of virtual training those few specialists delivering the remote training sessions could arrange further virtual sessions to discuss some of the barriers or issues encountered while performing actual patient manoeuvres.

### Benefits of Remote Training

In addition to general benefits such as the reduced travel for all participants and the ability to join from almost any location, aspects of this approach yield several other benefits which we also outline below, consistent with the Health and Care Professions Council training standards recognise that learning a new clinical skill requires theory and practical teaching, with support from experts (13). However, concerns have been highlighted on the quality of some online resources (14), which remains an important factor to consider with any such program development.

Key benefits include:

- The ability for pre-recorded content to be ready for delivery as needed.
- Possibility to scale to large number of pupils with reduced expenditure and a limited workforce of experts.
- An ability to ensure quality of content, once such a program has been validated. Importantly this avoids issues with free online content which can be excellent, however undecipherable to the nascent learner from poor content.

### Risk Mitigation

There exists a real concern that training may not provide the adequate competence for the practitioner to correctly complete the manoeuvre–in this instance what are primary concerns and how might they be mitigated? We consider two aspects–the first would be any associated risk to the patient, for example, the theoretical risk of arterial dissection in the neck, or perhaps displacing of crystals to adjacent semicircular canals. The second is incorrect diagnosis, both in terms of false positive and negative interpretation of results from the assessment manoeuvre. In both such instances however, this is not a problem exclusive to *remote* training, rather a consideration for all positional manoeuvre training. Moreover, theoretical risks of injury to the patient through neck “manipulation” are not evidenced in the real-life clinical setting, largely because these manoeuvres involve neck movements that lie well within physiological limits. Pillows can used for both the Dix-Hallpike and Epley manoeuvres that further mitigate this theoretical risk and such practical advice could be easily incorporated into remote learning sessions (15). Remote learning may in fact offer an opportunity to address and reduce such risks through repeated (virtual) exposure to correct techniques. Indeed, one recent study demonstrated basic surgical skills performance (which putatively holds greater risk) could be taught to medical students online with comparable levels of competency to conventional face to face teaching (16).

As with many bedside assessments and interventions undertaken by non-specialist staff, the positional manoeuvres we describe here involve a component of patient manual handling. Incidence of serious adverse events associated with performing them is exceptionally low, and any training program should include repeated practical sessions, education to provide an awareness of the risk and supervised performance when first testing patients.

Similarly, to improve clinical sensitivity and specificity, supervised performance of the novice can provide timely feedback when first testing patients and provide an environment for accelerated learning. In addition to this convention however, training resources such as those constituted in a remote program, readily accessible, could serve to further augment the accuracy of diagnosis, by presenting practice and virtual assessment scenarios for the non-specialist to revisit during skill acquisition. In any case, the constituent program we outline here focuses on training for the non-specialist with the aim of such training to enhance the assessment and identification and treatment of BPPV. We anticipate such a program would reduce the need for specialist review in many instances, however there will remain many occurrences which necessitate specialist referral. Box 4 provides examples when onward referral may be indicated, such guidance should form a central component of any non-specialist training, and should be adapted based on the setting and staff being trained.

Box 4 **Indications for onward referral.**

There are many causes of vertigo some of which can be life-threatening, such as stroke or tumour. In any instance if the non-specialist is unsure, urgent onward referral would be advised. This would extend to any patient history which includes recent head trauma or associated neurological symptoms in addition to vertigo (e.g. headache).

Vestibular migraine is another common cause of vertigo and can be correctly identified using specific diagnostic criteria. Dizzy patients who are assessed for, but do not have BPPV should be referred onward to a specialist capable of diagnosing vestibular migraine.

Dix-Hallpike test should be applied to any patient with brief episodic vertigo (and no spontaneous nystagmus). Patients that present with spontaneous (i.e. non-positional) or gaze evoked nystagmus require urgent onward referral.

Patients with a first attack of persistent vertigo and nystagmus.

In instances where nystagmus following a positional assessment manoeuvre is difficult to characterise, or the non-specialist is feels unable to correctly define the elicited nystagmus

Nystagmus difficult to characterise or define

Nystagmus atypical for posterior canal BPPV

Downbeat nystagmus

Upbeat nystagmus

Horizontal nystagmus^1^

Nystagmus spontaneously changes direction despite head being kept in the same position

## Conclusions

Recent evaluations during the COVID-19 pandemic of medical student experiences using digital learning and assessment platforms suggest the majority prefer conventional approaches to learning (17). However, this does not necessarily indicate these platforms are unsuccessful or could not make a large contribution to education in future. Current trends and changes to digital communication and technologies more generally, have seen an exponential increase in online resources. As a result, the leap toward telemedicine-based solutions is far more plausible than it has been previously.

As this relates to BPPV manoeuvre training, the greatest challenge is likely to be in the practical teaching to novice practitioners. There is however no specific reason such an approach is not possible and should not be tested and validated. If proven successful, the benefits extend much further than the immediate training itself. As is witnessed in other digital technologies, such a program may address one of the greatest challenges in BPPV training *per se*, the ability to standardise and scale the resource so that quality is maintained while having the capacity to reach a greater audience.

## Author Contributions

VT conceptualised the idea, wrote the preliminary manuscript, and approved the final version. AM conceptualised the idea, commented critically on the manuscript, and approved the final version. DK conceptualised the idea, wrote the preliminary manuscript, compiled the figure, and approved the final version. All authors listed have made a substantial, direct and intellectual contribution to the work, and approved it for publication.

## Conflict of Interest

The authors declare that the research was conducted in the absence of any commercial or financial relationships that could be construed as a potential conflict of interest.

## Publisher's Note

All claims expressed in this article are solely those of the authors and do not necessarily represent those of their affiliated organizations, or those of the publisher, the editors and the reviewers. Any product that may be evaluated in this article, or claim that may be made by its manufacturer, is not guaranteed or endorsed by the publisher.

## Footnotes

^1^horizontal canal BBPV would cause horizontal nystagmus but this is a relatively rare variant with between 5-30% of all BPPV cases.
